# Pretreatment Prediction of Adaptive Radiation Therapy Eligibility Using MRI-Based Radiomics for Advanced Nasopharyngeal Carcinoma Patients

**DOI:** 10.3389/fonc.2019.01050

**Published:** 2019-10-16

**Authors:** Ting-ting Yu, Sai-kit Lam, Lok-hang To, Ka-yan Tse, Nong-yi Cheng, Yeuk-nam Fan, Cheuk-lai Lo, Ka-wa Or, Man-lok Chan, Ka-ching Hui, Fong-chi Chan, Wai-ming Hui, Lo-kin Ngai, Francis Kar-ho Lee, Kwok-hung Au, Celia Wai-yi Yip, Yong Zhang, Jing Cai

**Affiliations:** ^1^Department of Health Technology and Informatics, Hong Kong Polytechnic University, Hung Hom, Hong Kong; ^2^Department of Clinical Oncology, Queen Elizabeth Hospital, Hong Kong, China; ^3^Department of Physics, Xiamen University, Xiamen, China

**Keywords:** radiomics, nasopharyngeal carcinoma, adaptive radiation therapy, tumor shrinkage, magnetic resonance imaging

## Abstract

**Background and purpose:** Adaptive radiotherapy (ART) can compensate for the dosimetric impacts induced by anatomic and geometric variations in patients with nasopharyngeal carcinoma (NPC); Yet, the need for ART can only be assessed during the radiation treatment and the implementation of ART is resource intensive. Therefore, we aimed to determine tumoral biomarkers using pre-treatment MR images for predicting ART eligibility in NPC patients prior to the start of treatment.

**Methods:** Seventy patients with biopsy-proven NPC (Stage II-IVB) in 2015 were enrolled into this retrospective study. Pre-treatment contrast-enhanced T1-w (CET1-w), T2-w MR images were processed and filtered using Laplacian of Gaussian (LoG) filter before radiomic features extraction. A total of 479 radiomics features, including the first-order (*n* = 90), shape (*n* = 14), and texture features (*n* = 375), were initially extracted from Gross-Tumor-Volume of primary tumor (GTVnp) using CET1-w, T2-w MR images. Patients were randomly divided into a training set (*n* = 51) and testing set (*n* = 19). The least absolute shrinkage and selection operator (LASSO) logistic regression model was applied for radiomic model construction in training set to select the most predictive features to predict patients who were replanned and assessed in the testing set. A double cross-validation approach of 100 resampled iterations with 3-fold nested cross-validation was employed in LASSO during model construction. The predictive performance of each model was evaluated using the area under the receiver operator characteristic (ROC) curve (AUC).

**Results:** In the present cohort, 13 of 70 patients (18.6%) underwent ART. Average AUCs in training and testing sets were 0.962 (95%CI: 0.961–0.963) and 0.852 (95%CI: 0.847–0.857) with 8 selected features for CET1-w model; 0.895 (95%CI: 0.893–0.896) and 0.750 (95%CI: 0.745–0.755) with 6 selected features for T2-w model; and 0.984 (95%CI: 0.983–0.984) and 0.930 (95%CI: 0.928–0.933) with 6 selected features for joint T1-T2 model, respectively. In general, the joint T1-T2 model outperformed either CET1-w or T2-w model alone.

**Conclusions:** Our study successfully showed promising capability of MRI-based radiomics features for pre-treatment identification of ART eligibility in NPC patients.

## Introduction

Due to the high proximity of the primary NPC tumor to the surrounding critical organs (spinal cord, brainstem, parotid glands) and metastatic neck lymph nodes, NPC is rarely treated surgically; radiation therapy (RT) remains the mainstay of NPC treatment (1). Intensity-modulated radiation therapy (IMRT) with/without induction chemotherapy (IC) or adjuvant chemotherapy (AC) is currently the standard of care for NPC patients (1). In clinical practice, RT treatment plans are tailor-made based on anatomic information of individual patients from their pre-treatment planning computed tomography (CT) images to maximize the radiation dose to tumor while protecting nearby critical structures and maintaining sufficiently high dose coverage to surrounding nodal targets.

However, an abundance of research has shown that significant anatomic and geometric variations are not uncommon throughout the course of RT for NPC due to either body weight loss (BW loss) or tumor regression (2–8). Radiation-induced mucositis is a common and debilitating complication for RT to HNC patients, which can lead to severe pain and difficulty in eating, largely affecting one's nutritional intake and resulting in significant BW loss. A prospective study reported a 37% of BW loss > 5 kg by the end of treatment (9). Patients having significant BW loss tends to accompany with reduced skin separation at various levels of cervical spine and neck (10), causing positional variability due to possible head movement inside the thermoplastic cast. Consequently, such variations would leave the issue of whether the contour deviations induced significant dose deviations in target or organs at risk. For tumor regression, Hu et al. (6) conducted a retrospective study and reviewed the planning CT and re-CT images of 40 re-planned NPC patients and confirmed the significant clinical-target-volume shrinkage of 35.1%. Murat et al. (11) also reported median percentage change in GTV of HNC patients for primary (26.8%), nodal (43.0%), and total (31.2%) GTVs. Indeed, when significant tumor shrinkage occurs, those critical organs might move into the original high dose region, leading to deleterious dosimetric impact on the surrounding organs (3, 4, 12) and/or insufficient dose delivery to targets (4, 13). ART can compensate for these dosimetric impact and maintain desirable therapeutic index. The clinical and dosimetric benefits of ART for HNC and NPC cancer patients have been widely reported (14–17). Yet, the implementation of ART is limited by several reasons. First, the choice to ART can be resource intensive and time-consuming for repeat imaging, re-contouring, re-planning, and analyzing dosimetric impacts between previous and new treatment plans, adding significant clinical burden and cost of patient care to an oncology center. Hence, performing ART on a patient basis is clinically impractical, especially for some busy units. Second, due to the nature of multifactorial ART eligibility, there is no universal selection protocol for ART that can be applied to all hospitals. In this regard, a huge amount of efforts has been constantly made to identify possible ART criteria for HNC and NPC cancer patients (5–7, 11, 18–21) to facilitate the clinical application of ART. Despite that, the current ART practice in most oncology centers, particularly for those busy units, is not efficient. The need for ART of each patient can now be only assessed during the RT treatment. Therefore, pre-treatment identification of high-risk NPC patients for ART is crucially favorable to achieve optimal personalized RT treatment, enabling radiation oncologists to more effectively and accurately prescribe ART for NPC patients and streamline resources management in clinical settings.

Recently, the field of radiomics together with rapid machine learning paradigms have increasingly gained popularity in the community of medical research, paving the way toward precision and personalized medicine (22). Radiomics, first introduced by Lambin et al. (22), is now shifting the role of medical imaging beyond the traditional diagnostic purposes. It allows for transformation of digitally encrypted medical images into mineable high-dimensional data, which can then be quantitatively analyzed to decode concealed genetic and molecular traits for decision making in oncology (23). While the predictive powers of radiomics in both cancer diagnosis and disease progression have been widely investigated (24–28), an extremely limited effort has yet been made to identify cancer patients for ART. Given the evidence of significant tumor shrinkage between two CT scans along RT treatment for re-planned NPC patients, we hypothesize that radiomic features extracted from 3-dimensional tumor volume contain predictive biomarkers for tumor shrinkage following cancer treatment—an implication for ART.

To our best knowledge, there is no research to include radiomics in predicting ART eligibility for NPC patients and its tumoral predictive biomarkers for ART has not been explored before. The objective of our study was to identify tumoral radiomic features using multi-parametric MR images, which are capable of predicting the ART eligibility for NPC patients. A study flow of current study is shown in Supplementary Figure 1.

## Methods and Materials

### A Predefined Hypothesis

Radiomic features extracted from 3-dimensional tumor volume contain predictive biomarkers for tumor shrinkage following cancer treatment—an implication for ART.

### Patients

#### Patient Source

**Figure 1:**
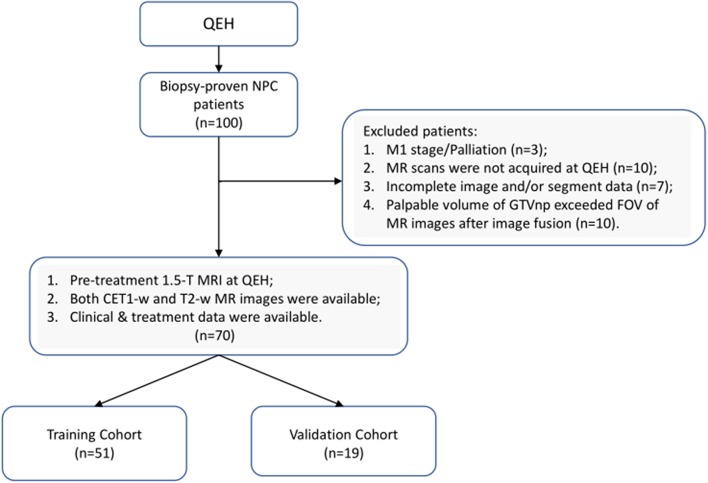
Inclusion and exclusion criteria used in the current study.

The current research was approved by the Human Subjects Ethics Sub-committee of the Hong Kong Polytechnic University and Kowloon Central/Kowloon East Cluster Research Ethics Committee of the Hospital Authority. This is a retrospective study, based on analyses of anonymized radiographic data and clinical data, the requirement for individual informed consent was waived. A total of 100 newly diagnosed patients with biopsy-proven (II-IVB) NPC (According to 7th edition of American Joint Committee on Cancer/Union for International Cancer Control TNM staging system) who received primary radiation therapy with/without chemotherapy at the Department of Clinical Oncology of Queen Elizabeth Hospital (QEH) between April 2015 and February 2016 were retrospectively reviewed. Based on the inclusion and exclusion criteria (IEC), 70 eligible patients were enrolled in the current study and randomly stratified into training (*n* = 51) and testing (*n* = 19) sets, as illustrated in Figure 1 (Details of the IEC is described in Supplementary Material).

#### Patient Data

Patient clinical data, including demographic information (age, gender) and tumor characteristics (T stage, N stage, histological subtype); imaging data (planning CT images, pre-treatment CET1-w and T2-w MR images); treatment-related data (contouring data, treatment machine, treatment strategies, dose fractionation scheme); outcome data (re-plan status and any replan-related medical records) were retrospectively collected.

#### Treatment

In general, patients with early-stage (I-II, *n* = 3) tumors were treated with curative RT alone, while those with advanced-stage (III-IVB, *n* = 67) were treated with radical concurrent chemoradiotherapy (CCRT), with/without IC or AC. Pre -treatment MRI and planning CT scans were performed within a week prior to the start of IC treatment for target delineation and during the last cycle of IC treatment, respectively. In our dataset, 7 out of 70 patients received IC, while only one underwent ART procedures, who subsequently refused further IC after completion of the first cycle due to repeated vomiting. See Supplementary Material for details of the chemotherapy and RT regimen.

#### Clinical Endpoint

The clinical endpoint of this study was defined as the re-plan status of patients: whether or not a patient received ART during RT treatment at the discretion of radiation oncologist.

### Multifactorial ART Eligibility

A daily megavoltage CT (MVCT) or cone beam CT (CBCT) or planar orthogonal X-rays was taken for all patients to correct for positional variations and to assess anatomic or geometric changes throughout the entire treatment chain. Additionally, weekly records of body weight were made to assess whether significant body weight loss (BWL > 10%) occurred.

The Radiation Oncology team reviewed daily scans on a regular basis, considering BWL of individual patients. When BWL > 10% occurred, possibly accompanied with noted change in body or neck contour, significant lymph nodes regression and/or loss of neck tissue, an adaptive review process was initiated, where the original plan was re-calculated on the MVCT scan for initial dosimetric evaluation to determine whether further actions (re-CT and/or re-plan) or continuous monitoring were appropriate. Patients who did not receive any actions from the first review session were then proceeded with original plan until the next review session for another dosimetric evaluation. On plan review, radiation oncologist assessed the geometric, volumetric and dosimetric variations of both target and organs at risk (OARs) structures through both visual inspection and dosimetric evaluation. The decision to generate a re-plan was at the discretion of the treating radiation oncologist. Considerations influencing ART implementation included risks of insufficient primary and nodal targets coverage, overdose to critical organs (such as spinal cord, optic chiasm, and brainstem), increase of high skin dose areas over neck, and unfit of thermoplastic cast for patient immobilization.

**Figure 2:**
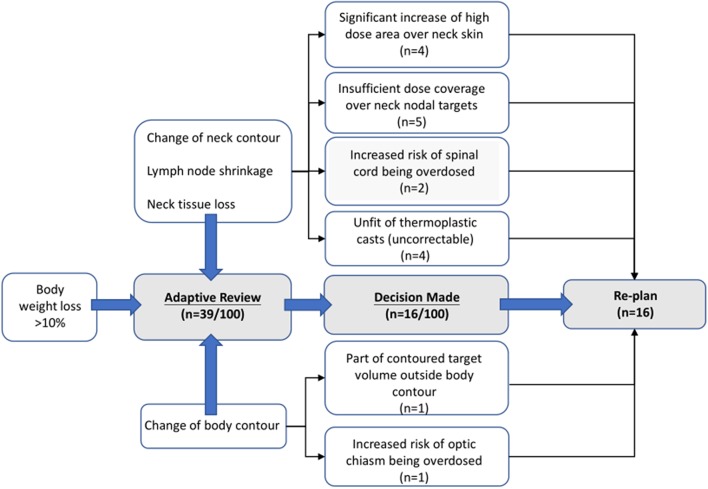
An illustrative example of clinical decision on ART implementation.

In our dataset, 39 (of 100) patients were initially enrolled into the adaptive review processes, while only 16 ultimately received re-planned procedures. Among the 16 patients, 13 were enrolled in our study, the replans were mostly done during week 4–5 and after the 20th fraction on average A diagram of leading causes for ART implementation are illustrated in Figure 2. A detailed qualitative summary of how those 39 patients were screened and selected can be found in Supplementary Material.

### MRI Acquisition and Segmentation

All 70 patients were scanned with 1.5-T MRI (Avanto, Siemens, Germany) at QEH. We acquired T2-w and CET1-w Digital Imaging and Communications in Medicine (DICOM) images archived using Picture Archiving and Communication System (PACs). The MR images acquisition parameters can be found in Supplementary Material. Intravenous contrast enhanced computed tomography (CT) simulation was performed at 3 mm intervals from the vertex to 5 cm below the sternoclavicular notch with a 16-slice Brilliance Big Bore CT (Philips Medical Systems, Cleveland, OH). All segmentations (tumor, nodal volume and other organs-at-risk) were manually delineated on axial CT slices by an experienced radiation oncologist (with >20 years of experience), which was then fused with MR images for further processing.

### MRI Image Preprocessing

Before extracting radiomic features, all MR images were processed using 3DSlicer (version 4.11.0). Isotropic resampling was performed by linear interpolation to obtain a voxel size of 1 × 1 × 1 mm to account for variations in scanning parameters between studied MR series. MRI inhomogeneity correction was applied to account for the locally varying intensity using N4ITK algorithm. To ensure meaning comparison of the extracted features values across all patients, intensity normalization was conducted using brainstem as a reference ROI, which was chosen because its signal intensity is comparatively homogeneous. The existing contour of the brainstem structure for RT planning purpose was modified to exclude air. Image discretization with a fixed bin width of 5 to maintain constant intensity resolution across resampled images. Apart from the original images, image reconstructions were performed using Laplacian of Gaussian (LoG) filter with sigma values of 2, 3, 4, 5 mm to extract features at multiple scales of resolution, from fine, medium to coarse.

### Feature Extraction and Preprocessing

**Figure 3:**
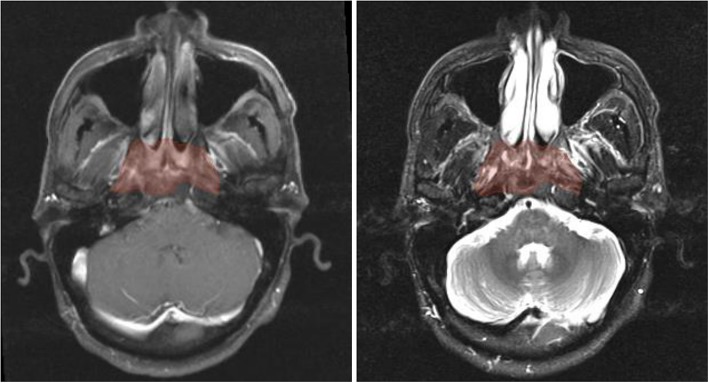
Axial pre-treatment morphological MR images of a 44-year-old man with undifferentiated carcinoma of NPC (T3N2M0). Features of radiomics were extracted from the primary tumor area -GTVnp (red overlay). From left to right: CET1-w and T2-w MR image, respectively.

A total of 479 radiomic features were extracted from GTVnp on CET1-w and T2-w MR images, respectively, using SlicerRadiomics in 3D Slicer (version 4.11.0). A representative example of axial pre-treatment MR images with GTVnp contour is shown in Figure 3. Extracted features included shape features (*n* = 14), first-order intensity features (*n* = 90), and texture features (*n* = 375) (See Supplementary Material for a detailed listing of extracted features). All extracted radiomics features were centered and scaled to a value with a mean of 0 and a standard deviation of 1 (z-score transformation) before further analysis using R software (version 3.5.2).

### Feature Selection and Model Optimization Methodology

To avoid over sensitive model, we removed highly inter-correlated radiomics features. By using the R package “caret,” we computed Pearson correlation coefficient (PCC) based on a correlation matrix to quantify the pair-wise correlations. If two radiomic features appeared a strong correlation with an absolute correlation coefficient (*r*) ≥ 0.9, we removed the feature with the largest mean absolute correlation. As a result, we obtained a primary feature set of 53 from 479.

**Figure 4:**
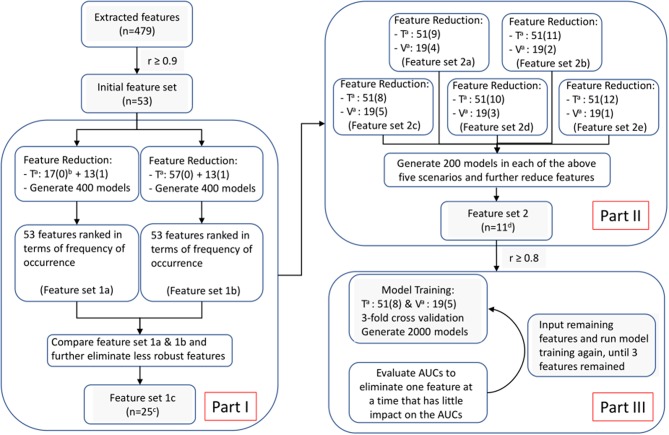
Feature selection and model optimization methodology. Superscript “a”: T for training cohort; V for validation cohort. “b”: The number inside the parentheses is either “1” or “0,” representing “re-planned” and “not re-planned” patients; Numbers in front of the parentheses indicate number of patients. “c”: 25 features remained in feature set 1c for CET1-w-based model; while 28 and 39 for T2-w-based and Joint T1-T2-based models, respectively. “d”: 16 features remained in feature set 2 for CET1-w-beased model; while 13 and 22 for T2-w-based and Joint T1-T2-based models, respectively.

Following this, we applied Least Absolute Shrinkage and Selection Operator (LASSO) algorithm in R package “glmet” to select the most predictive radiomic features based on the ART status of patients in the training set. The LASSO is typically applied to select high-dimensional biomarkers, and coefficients of the regression variables were penalized in the process of regularization to minimize the prediction error. The ratio of patients who did not receive ART (*n* = 57) to those who did (*n* = 13) was 4, approximately. Considering the imbalance data, we adopted our three-step feature screening strategy, as illustrated in Figure 4, to construct CET1-w, T2-w, and joint T1-T2 based radiomic models. The first two steps aimed to further eliminate less/least predictive features in terms of their frequency of occurrence among hundreds of generated models. With the reduced features, we performed PCC with *r* ≥ 0.8 to avoid highly correlated features in our final models. Lastly, model trainings were performed with reduced number of input features using a double cross-validation approach, similar to the one adopted by Xu et al. (29) In short, 100 random sampling was conducted to balance the class distribution within the cross-validation partitions, which would result in a distribution of AUC values across the generated models and hence allow us to assess the model performance. A 3-fold nested cross-validation was performed with 20 repetition to determine the optimal value for the model tuning parameter (λ). As a result, a total of 2,000 models were generated for each input set of features (See Supplementary Material for feature screening methodology). In total, 8 sets of radiomic features with number of variables ranging from 3 to 10 were analyzed for the prediction capability in terms of AUCs using box and whisker plots and 95 percent confidence interval (CI).

### Statistical Analysis

The statistical correlations between available clinical data and replan status were assessed using univariate logistic regression. All statistical analyses were performed using R software (version 3.5.2). The following R packages were used: The glmnet package was used for LASSO logistic regression. The caret package was used to perform Pearson correlation study. The ROCR package was employed to perform ROC analysis. All statistical tests were two-sided, and *P*-values of <0.05 were considered significant.

## Results

The demographic and tumor characteristics of 70 NPC patients are summarized in Table 1. Thirteen (18.6%) patients who underwent ART procedure were included. There is no statistical association between the available clinical data and re-plan incidence.

**Table 1:** Patient characteristics in the present cohort.

**Clinical factor**	**Category**	**Number (Percent)**	***P*-values**
Gender	Male	50 (71.4%)	0.2558
	Female	20 (28.6%)	
Age in years	<51	21 (30%)	0.386
	51–70	42 (60%)	
	>70	7 (10%)	
T stage	T1	2 (2.9%)	0.554
	T2	2 (2.9%)	
	T3	50 (71.4%)	
	T4	16 (22.8%)	
N stage	N1	5 (7.1%)	0.859
	N2	56 (80%)	
	N3	9 (12.9%)	
Overall stage	Stage II	3 (4.3%)	0.535
	Stage III	43 (61.4%)	
	Stage IV	24 (34.3%)	
Histology	Type I	3 (4.3%)	0.827
	Type II	1 (1.4%)	
	Type III	66 (94.3%)	
Treatment	EBRT-alone	14 (20%)	0.8411
	CCRT	37 (52.9%)	
	CCRT + AC	11 (15.7%)	
	IC + CCRT	7 (10%)	
	Others	1 (1.4%)	
Initial weight (kg) (average ± SD)	Replan Group	61.6 ± 15.5	0.929
	Non-replan Group	61.9 ± 12.2	

*EBRT, External Beam Radiation Treatment; CCRT, Concurrent Chemotherapy Radiation Treatment; IC, Induction Chemotherapy; AC, Adjuvant Chemotherapy; Type I, Keratinizing squamous cell carcinoma; Type II, Non-keratinizing differentiated carcinoma; Type III, Non-keratinizing undifferentiated carcinoma*.

**Figure 5:**
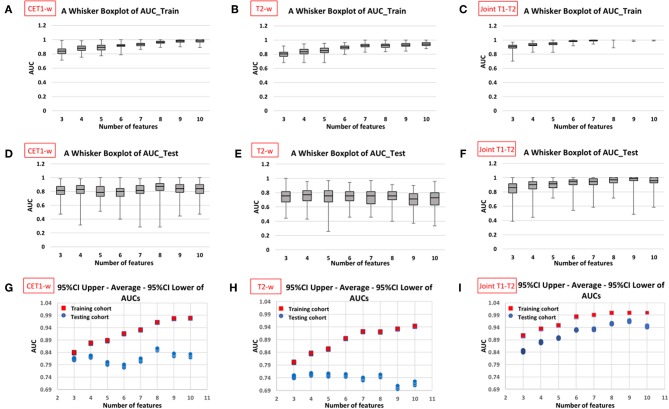
Distribution of AUC values in training and testing cohorts against different number of features in the constructed models from 100 resampled iterations of 20-repeated 3-fold cross validation (CET1-w model: first column, T2-w model: second column, and Joint T1-T2 model: third column). The box and whisker plots in first **(A–C)** and second rows **(D–F)** display the AUC distributions with varying number of selected features in training cohort and testing cohort, respectively; the plots in third row **(G–I)** displays 95% confidence interval and average AUCs for both cohorts against number of selected features in the models.

Figure 5 displays the AUC distributions for each feature set (from 3 to 10 features). Figures 5A–C shows the box and whisker plots of the three types of models (CET1-w, T2-w, and joint T1-T2) for training set; Figures 5D–F are for testing set; Figures 5G–I visualizes the range of 95% CI of AUCs in both training and testing sets for the three types of models. The optimal feature sets for each type of models were determined considering the overall distribution of AUC values and its stability. When adding one more feature to the current feature set made no/less difference to the AUC values, the current feature set was considered as the optimal feature set that would give optimal predictive performance of our models. Selected features for each model are listed in Table 2.

**Table 2:** Table of selected features in CET1-w, T2-w, and joint T1-T2 radiomics models.

**MRI series**	**Selected radiomics features**	**CET1-w model**	**T2-w model**	**Joint T1-T2 model**
CET1-w	Original shape Sphericity	✓		
CET1-w	Original shape Maximum 2D Diameter Slice	✓		
CET1-w	Log-sigma-2-0-mm-3D glcm MCC	✓		✓
CET1-w	Log-sigma-2-0-mm-3D first-order Kurtosis	✓		✓
CET1-w	Log-sigma-3-0-mm-3D first-order Skewness	✓		✓
CET1-w	Log-sigma-4-0-mm-3D first-order Kurtosis	✓		
CET1-w	Log-sigma-5-0-mm-3D gldm Dependence Entropy	✓		
CET1-w	Log-sigma-5-0-mm-3D gldm Small Dependence Low Gray Level Emphasis	✓		✓
CET1-w	Original first-order Kurtosis			✓
T2-w	Original shape Sphericity		✓	
T2-w	Original shape Elongation		✓	
T2-w	Log-sigma-2-0-mm-3D gldm Large Dependence High Gray Level Emphasis		✓	
T2-w	Log-sigma-2-0-mm-3D glcm Imc1		✓	
T2-w	Log-sigma-3-0-mm-3D ngtdm Strength		✓	
T2-w	Log-sigma-5-0-mm-3D first-order Kurtosis		✓	
T2-w	Log-sigma-3-0-mm-3D glcm Idn			✓

Average AUC values in training and testing sets were 0.962 (95%CI: 0.961–0.963) and 0.852 (95%CI: 0.847–0.857) with 8 selected features for CET1-w model; 0.895 (95%CI: 0.893–0.896) and 0.750 (95%CI: 0.745–0.755) with 6 selected features for T2-w model; and 0.984 (95%CI: 0.983–0.984) and 0.930 (95%CI: 0.928–0.933) with 6 selected features for joint T1-T2 model, respectively.

## Discussion

We successfully revealed the predictive capability of MRI-based radiomics in ART eligibility using our dataset. Eight features were identified for CET1-w model, including 2 shape features (sphericity, maximum 2D diameter slice) and 6 LoG-based features which include 3 first-order features (kurtosis, skewness) and 3 texture features (GLCM and GLDM). Six features were selected for T2-w model, including 2 shape features (sphericity, elongation) and 4 LoG-based features which include 1 first-order feature (kurtosis) and 3 texture features (GLDM, NGTDM). Six features were chosen for joint T1-T2 model, including 1 first-order feature (kurtosis) and 5 LoG-based features which consist of 2 first-order features (kurtosis, skewness) and 3 texture features (GLCM, GLDM), as shown in Table 2. With these selected features, we achieved average AUCs of 0.962 (0.852), 0.895 (0.750), 0.904 (0.930) in training (testing) set for CET1-w, Tw-2 and joint T1-T2 models, respectively, representing a promising result for pre-treatment prediction of ART eligibility in NPC patients.

Multiple groups have confirmed that significant tumor shrinkage occurs during RT, triggering the need for ART. Hu et al. (6) reviewed the planning CT and re-CT images of 40 re-planned NPC patients and confirmed the significant clinical-target-volume shrinkage of 35.1%. Murat et al. (11) reported median percentage change in GTV of HNC patients for primary (26.8%), nodal (43.0%), and total (31.2%) GTVs. Lee H et al. confirmed average volume reduction of GTVnp of 45.9 cm^3^ (pre-RT) to 26.7 cm^3^ (third week of RT) in 159 NPC patients. All these studies have suggested that tumor shrinkage serves as a favorable ART criterion. However, only a few studies have developed ART selection strategies based on the tumor volume reduction. Murat et al. (11) developed a decision tree for tumor shrinkage for HNC patients, incorporating initial target volumes and other clinical factors; although an accuracy of 88% was reported in predicting the tumor shrinkage in 48 patients, the validity was not tested and some of the clinical factors used may not be available in other clinics, such as tumor growth pattern (endophytic or exophytic), hindering the generalizability of the decision tree. Recently, Ramella et al. (30) explored the radiomic capability for ART in lung cancer patients and reported that radiomic features extracted from planning target volume (PTV) of lung cancer on CT images were capable of distinguishing patients between ART and non-ART group with AUC of 0.82, on the ground of tumor shrinkage during treatment. To our best knowledge, this study is the first to include radiomics in predicting ART eligibility for NPC patients and its tumoral predictive biomarkers for ART has not been explored before. Our promising results are also in line with the work done by Ramella et al. (30)

In our experience, we observed that the joint T1-T2 radiomic model outperformed either CET1-w or T2-w alone model in terms of AUCs in both training and testing sets. From Figures 5G–I, it can be observed that the joint T1-T2 model gives a more consistent variation in 95% CI of AUCs against different feature sets in both training and testing sets, suggesting that joint T1-T2 model might be the preferable predictive system among the others. Another interesting observation was that the majority (5 of 6) of the selected features in the joint T1-T2 model were from CET1-w images, suggesting that features from CET1-w images might be more predictive than those from T2-w images. A possible reason could be attributed to the inherent limitation of LASSO; when pairwise correlations exist between predictors, the LASSO picks one correlated predictor and ignores the rest. To account for this, we performed another PCC with *r* ≥ 0.8 prior to part III in our feature selection methodology (Figure 4) to avoid highly correlated features in our final models. Further investigations on the feature selection methodology will be part of our future studies.

On the other hand, NPC radiomics studies on MR images have been widely studied, focusing mainly on prediction of prognosis (disease progression) and treatment response to either induction chemotherapy (IC) or chemo-radiotherapy, while prediction of the need for replanning has not been previously reported. Besides, each study developed a unique radiomic signature for the same outcome prediction, which limits the feasibility to directly compare all the resultant features between studies. However, interestingly, categories of resultant features might be different depending on prediction outcomes, which might explain our results to some extent. For prognostic prediction, texture features were obviously dominant in their final radiomic signatures relative to first-order and shape features, and GLCM (Gray-Level Co-occurrence Matrix) was the only shared-feature category between studies. A possible rationale might be that the texture features were considered to reflect intra-tumor heterogeneity by depicting the spatial arrangement of voxels (regularity) and variability of local intensity within tumor, which was acknowledged as a characteristic of malignancy. For prediction of treatment response, while GLCM were still the only common resultant feature category between studies, however, first-order features were dominant in final radiomics signature. Wang et al. investigated the capability of MRI-based radiomic signatures to predict early response to IC for NPC patients using T1-w, CET1-w, and T2-w MR images. Among the 15 features selected in their joint-T1-CET1-T2-w model, 7 were first-order features, three were GLCM features, and the rest were Gabor and wavelet features. Another radiomic study by Hou et al. (31) exploring feasibility of CECT-based biomarkers to predict therapeutic response of esophageal carcinoma to chemo-radiotherapy reported that first-order features (skewness and/or kurtosis) were identified as significant parameters for differentiating SDs (stable disease) from PRs (partial response) and SDs from CRs (complete response). In both studies, the tumor response was assessed according to the Response Evaluation Criteria in Solid Tumors (RECIST), which takes into account the reduction of tumor size following treatment. Similar to our study, we hypothesized that the image-based tumoral biomarkers are predictive to tumor shrinkage.

In our results, shape features (e.g., Sphericity, Elongation, Maximum 2D diameter slice) and/or first-order features (e.g., kurtosis and skewness) were generally dominant relative to texture features in our models, which is consistent with results from abovementioned radiomic studies for treatment response prediction. Interestingly, kurtosis and/or skewness and GLCM-based features are the common features shared in all three models. Kurtosis and skewness measure the peakiness and asymmetry of the histogram, respectively, while GLCM features quantify the spatial gray-level variation within local neighbors on a pixel basis. Nevertheless, the understanding of the meaningfulness of these features, especially in relation to the prediction outcome, is still largely unknown and deserves further investigations.

This study has several limitations. Firstly, the heterogeneity of image acquisition and reconstruction protocols and ART strategies in different medical centers limit the generalizability of the identified models and reproducibility of the selected features. In future study, we will perform testing on different datasets obtained from other oncology departments with patients undergoing MRIs on different scanners. Secondly, the rate of adaptive replannings in the small cohort is relatively low. A more convincible conclusion could be drawn by recruiting larger cohorts with more balanced dataset between patients who underwent replan and those did not, which will be part of our future efforts. Lastly, the retrospective nature of this study might account for the potential bias. However, the novelty of this study was to highlight the capability of using pre-treatment MRI radiomic features to predict which patients undergoing radiotherapy for NPC were selected for ART. In future study, radiomics features from other ROIs and other pertinent non-radiomic clinical data, such as volumetric and dosimetric data of tumor and nearby organs (e.g., lymph nodes and parotid glands), and geometric relations among these structures, will be incorporated into our radiomics models in future to yield a more comprehensive prediction.

## Conclusion

The present study successfully demonstrated promising capability of MRI-based radiomics for pre-pretreatment identification of ART eligibility in NPC patients. In particular, the joint T1-T2 model with 6 selected radiomic features appears to be the preferable predictive system over other studied models. This would allow radiation oncologists to more effectively and accurately prescribe ART on individual patient basis to achieve true personalized radiotherapy for NPC patients, meanwhile streamlining resources management in clinical settings. In future work, multi-institution prospective studies with larger patient sample are warranted to improve the clinical efficacy of our models.

## Data Availability Statement

The datasets generated for this study are available on request to the corresponding author.

## Ethics Statement

The studies involving human participants were reviewed and approved by Human Subjects Ethics Sub-committee of the Hong Kong Polytechnic University and Kowloon Central/Kowloon East Cluster Research Ethics Committee of the Hospital Authority. Written informed consent for participation was not required for this study in accordance with the national legislation and the institutional requirements.

## Author Contributions

YZ, KA, FL, JC, TY, and SL contributed to study design, methodology development, results interpretation, and manuscript review. CY offered administrative and material support for data collection. SL, TY, MC, KT, NC, YF, CL, KO, LT, KH, FC, WH, and LN collected patients clinical and imaging data. MC, KT, NC, YF, CL, KO, LT, KH, FC, and WH contributed to the image preprocessing and feature extraction. TY constructed the models. SL wrote the manuscript. JC supervised the study.

### Conflict of Interest

The authors declare that the research was conducted in the absence of any commercial or financial relationships that could be construed as a potential conflict of interest.

## References

[1] Ng WT Wong ECY Lee VHF Chan JYW Lee AWM. Head and neck cancer in Hong Kong. Jpn J Clin Oncol. (2018) 48:13–21. 10.1093/jjco/hyx151 29145620

[2] Cheng HC Wu VW Ngan RK Tang KW Chan CC Wong KH. A prospective study on volumetric and dosimetric changes during intensity-modulated radiotherapy for nasopharyngeal carcinoma patients. Radiother Oncol. (2012) 104:317–23. 10.1016/j.radonc.2012.03.013 22551564

[3] Jin X Han C Zhou Y Yi J Yan H Xie C. A modified VMAT adaptive radiotherapy for nasopharyngeal cancer patients based on CT-CT image fusion. Radiat Oncol. (2013) 8:277. 10.1186/1748-717X-8-277 24279414 PMC4222034

[4] Zhao L Wan Q Zhou Y Deng X Xie C Wu S. The role of replanning in fractionated intensity modulated radiotherapy for nasopharyngeal carcinoma. Radiother Oncol. (2011) 98:23–7. 10.1016/j.radonc.2010.10.009 21040992

[5] Lee H Ahn YC Oh D Nam H Noh JM Park SY. Tumor volume reduction rate during adaptive radiation therapy as a prognosticator for nasopharyngeal cancer. Cancer Res Treat. (2016) 48:537–45. 10.4143/crt.2015.081 26194371 PMC4843740

[6] Hu YC Tsai KW Lee CC Peng NJ Chien JC Tseng HH. Which nasopharyngeal cancer patients need adaptive radiotherapy? BMC Cancer. (2018) 18:1234. 10.1186/s12885-018-5159-y 30526538 PMC6288867

[7] Tan W Li Y Han G Xu J Wang X Li Y. Target volume and position variations during intensity-modulated radiotherapy for patients with nasopharyngeal carcinoma. Oncotargets Ther. (2013) 6:1719–28. 10.2147/OTT.S53639 24311943 PMC3839809

[8] Barker JL Jr Garden AS Ang KK O'Daniel JC Wang H Court LE. Quantification of volumetric and geometric changes occurring during fractionated radiotherapy for head-and-neck cancer using an integrated CT/linear accelerator system. Int J Radiat Oncol Biol Phys. (2004) 59:960–70. 10.1016/j.ijrobp.2003.12.024 15234029

[9] Munshi A Pandey MB Durga T Pandey KC Bahadur S Mohanti BK. Weight loss during radiotherapy for head and neck malignancies: what factors impact it? Nutr Cancer. (2003) 47:136–40. 10.1207/s15327914nc4702_5 15087265

[10] Noble DJ Yeap PL Seah SYK Harrison K Shelley LEA Romanchikova M. Anatomical change during radiotherapy for head and neck cancer, and its effect on delivered dose to the spinal cord. Radiother Oncol. (2019) 130:32–8. 10.1016/j.radonc.2018.07.009 30049455 PMC6358720

[11] Surucu M Shah KK Mescioglu I Roeske JC Small W Jr Choi M. Decision trees predicting tumor shrinkage for head and neck cancer: Implications for adaptive radiotherapy. Technol Cancer Res Treat. (2016) 15:139–45. 10.1177/1533034615572638 25731804

[12] Hansen EK Bucci MK Quivey JM Weinberg V Xia P. Repeat CT imaging and replanning during the course of IMRT for head-and-neck cancer. Int J Radiat Oncol Biol Phys. (2006) 64:355–62. 10.1016/j.ijrobp.2005.07.957 16256277

[13] Bhide SA Davies M Burke K McNair HA Hansen V Barbachano Y. Weekly volume and dosimetric changes during chemoradiotherapy with intensity-modulated radiation therapy for head and neck cancer: a prospective observational study. Int J Radiat Oncol Biol Phys. (2010) 76:1360–8. 10.1016/j.ijrobp.2009.04.005 20338474

[14] Schwartz DL Garden AS Shah SJ Chronowski G Sejpal S Rosenthal DI. Adaptive radiotherapy for head-and-neck cancer - dosimetric results from a prospective clinical trial. Radiother Oncol. (2013) 106:80–84. 10.1016/j.radonc.2012.10.010 23369744

[15] Schwartz DL Garden AS Thomas J Chen Y Zhang Y Lewin J. Adaptive radiotherapy for head-and-neck cancer: initial clinical outcomes from a prospective trial. Int J Radiat Oncol Biol Phys. (2012) 83:986–93. 10.1016/j.ijrobp.2011.08.017 22138459 PMC4271827

[16] Chen AM Daly ME Cui J Mathai M Benedict S Purdy JA. Clinical outcomes among patients with head and neck cancer treated by intensity-modulated radiotherapy with and without adaptive replanning. Head Neck. (2014) 36:1541–6. 10.1002/hed.23477 23996502

[17] Yang H Hu W Wang W Chen P Ding W Luo W. Replanning during intensity modulated radiation therapy improved quality of life in patients with nasopharyngeal carcinoma. Int J Radiat Oncol Biol Phys. (2013) 85:e47–54. 10.1016/j.ijrobp.2012.09.033 23122981

[18] Wu Q Chi Y Chen PY Krauss DJ Yan D Martinez A. Adaptive replanning strategies accounting for shrinkage in head and neck IMRT. Int J Radiat Oncol Biol Phys. (2009) 75:924–32. 10.1016/j.ijrobp.2009.04.047 19801104

[19] Fung WW Wu VW Teo PM. Developing an adaptive radiation therapy strategy for nasopharyngeal carcinoma. J Radiat Res. (2014) 55:293–304. 10.1093/jrr/rrt103 23988444 PMC3951067

[20] Brown E Owen R Harden F Mengersen K Oestreich K Houghton W. Adaptive radiotherapy: Predicting the need for adaptive radiotherapy in head and neck cancer. Radiother Oncol. (2015) 116:57–63. 10.1016/j.radonc.2015.06.025 26142268

[21] Brouwer CL Steenbakkers RJ Langendijk JA Sijtsema NM. Identifying patients who may benefit from adaptive radiotherapy: Does the literature on anatomic and dosimetric changes in head and neck organs at risk during radiotherapy provide information to help? Radiother Oncol. (2015) 115:285–94. 10.1016/j.radonc.2015.05.018 26094076

[22] Lambin P Rios-Velazquez E Leijenaar R Carvalho S van Stiphout RG Granton P. Radiomics: extracting more information from medical images using advanced feature analysis. Eur J Cancer. (2012) 48:441–6. 10.1016/j.ejca.2011.11.036 22257792 PMC4533986

[23] Lambin P Leijenaar RTH Deist TM Peerlings J de Jong EEC van Timmeren J. Radiomics: the bridge between medical imaging and personalized medicine. Nat Rev Clin Oncol. (2017) 14:749–62. 10.1038/nrclinonc.2017.141 28975929

[24] Zhang B Tian J Dong D Gu D Dong Y Zhang L. Radiomics features of multiparametric MRI as novel prognostic factors in advanced nasopharyngeal carcinoma. Clin Cancer Res. (2017) 23:4259–69. 10.1158/1078-0432.CCR-16-2910 28280088

[25] Zhang B He X Ouyang F Gu D Dong Y Zhang L. Radiomic machine-learning classifiers for prognostic biomarkers of advanced nasopharyngeal carcinoma. Cancer Lett. (2017) 403:21–7. 10.1016/j.canlet.2017.06.004 28610955

[26] Zhang B Ouyang F Gu D Dong Y Zhang L Mo X. Advanced nasopharyngeal carcinoma: pre-treatment prediction of progression based on multi-parametric MRI radiomics. Oncotarget. (2017) 8:72457–65. 10.18632/oncotarget.19799 29069802 PMC5641145

[27] Ouyang FS Guo BL Zhang B Dong YH Zhang L Mo XK. Exploration and validation of radiomics signature as an independent prognostic biomarker in stage III-IVb nasopharyngeal carcinoma. Oncotarget. (2017) 8:74869–79. 10.18632/oncotarget.20423 29088830 PMC5650385

[28] Dong D Tang L Li ZY Fang MJ Gao JB Shan XH. Development and validation of an individualized nomogram to identify occult peritoneal metastasis in patients with advanced gastric cancer. Ann Oncol. (2019) 30:431–8. 10.1093/annonc/mdz001 30689702 PMC6442651

[29] Xu CJ van der Schaaf A Van't Veld AA Langendijk JA Schilstra CXu CJ, van dSA, van't VA, Langendijk JA, Schilstra C. Statistical validation of normal tissue complication probability models. Int J Radiat Oncol Biol Phys. (2012) 84:e123–e129. 10.1016/j.ijrobp.2012.02.022 22541961

[30] Ramella S Fiore M Greco C Cordelli E Sicilia R Merone M. A radiomic approach for adaptive radiotherapy in non-small cell lung cancer patients. PLoS ONE. 13:e0207455. 10.1371/journal.pone.0207455 30462705 PMC6248970

[31] Hou Z Ren W Li S Liu J Sun Y Yan J. Radiomic analysis in contrast-enhanced CT: predict treatment response to chemoradiotherapy in esophageal carcinoma. Oncotarget. (2017) 8:104444–54. 10.18632/oncotarget.22304 29262652 PMC5732818

