# Supplementary material for: Pretreatment Prediction of Adaptive Radiation Therapy Eligibility Using MRI-Based Radiomics for Advanced Nasopharyngeal Carcinoma Patients
Source: Front Oncol. 2019 Oct 16;9:1050. doi: 10.3389/fonc.2019.01050 (PMC6805774; doi:10.3389/fonc.2019.01050)
Supplement: Supplementary file 1 [file Data_Sheet_1.PDF]

Supplementary Material

A study flow of current project

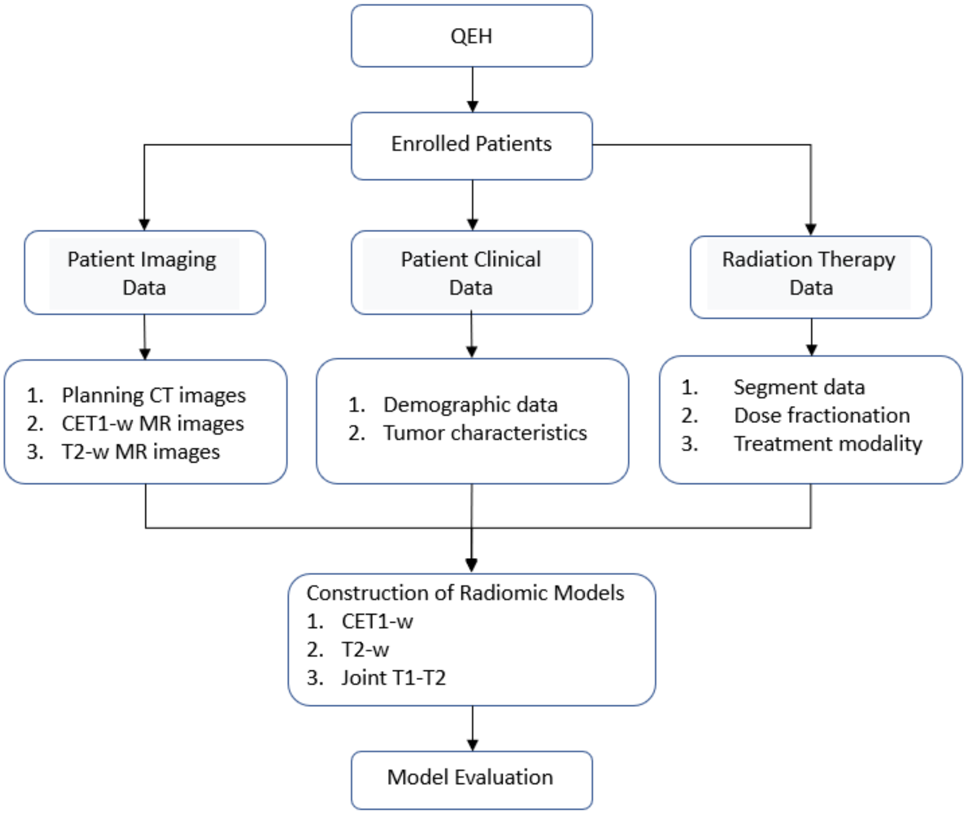

Supplementary Figure S1. An illustration of study flow of current study

Patient

Inclusion and exclusion criteria

Patients treated at QEH were included in this study if the following inclusion criteria were met: (a) evidence of biopsy-proven NPC; (b) availability of pre-treatment 1.5 telsa (1.5 T) MRI data acquired following the imaging protocol used at QEH; (c) availability of both Contrast-enhanced T1-weitghted (CET1-w) and T2-weighted (T2-w) MR images; (d) availability of clinical (e.g. age, gender, TNM stage, body weight data), treatment and outcome (re-plan status) data. Patients were excluded if any of the following exclusion criteria were met: (a) any evidence of distant metastasis at diagnosis prior to the initial treatment; (b) palliative treatment was intended; (c) MRI data acquired at hospitals/clinics other than QEH; (d) incomplete image data or segment data; (d) palpable volume of primary tumor (GTVnp) exceeded the field-of-view of MR images after image registration with planning CT images. Based on these criteria, a total of 70 patients were enrolled in this research. Eligible patients were randomly stratified into training (n = 51) and testing (n = 19) cohorts.

Treatment

The concurrent chemoradiotherapy regimen comprised cisplatin (30 mg/m^2 on a weekly basis, 3-5 cycles). Induction chemotherapy and adjuvant chemotherapy consisted of 3-week cycles of gemcitabine and cisplatin (GP) for 3 cycles and 4-week cycles of cisplatin and fluorouracil (PF4) for 3 cycles, respectively. Reasons for not receiving chemotherapy included age, organ dysfunction indicating intolerance to treatment, and an individual patient's refusal.

Intensity-modulated radiation therapy (IMRT) or TomoTherapy was administrated with a standard dose-fractionation schedule of 66 Gy in 33 fractions in 5 – 6 fractions/week to high-risk clinical target volume (CTV) with 3-mm margins in both nasopharynx (Planning target volume, PTVnp66) and neck region (PTVn66) with fractional dose of 2.18 Gy; Whereas, 60 Gy was prescribed to the low-risk CTV with 3-mm margins in both PTVnp60 and PTVn60 with 1.82 Gy/fraction; A simultaneous integrated boost (SIB) of 70 Gy was given to GTVnp with 3-mm margins (PTVnp70) to achieve optimal local tumor control with 2.12 Gy/fraction.

ART screening and selection

A retrospective review of 100 NPC patients who were treated with IMRT in 2015 and 2016 at QEH found that 39 patients were enrolled into a review of need for ART, among which only 16 patients were determined to receive a modification on original treatment, i.e. ART. Below is a qualitative summary of how those patients were screened and selected for the need of ART based on the existing dataset.

Among the 39 patients who were enrolled into a review of need for ART, the majority of patients (29/39, among which 14/29 patients subsequently underwent ART) were reviewed for the need of ART because of significant body weight (BW) loss (>10% drop from the initial body weight measured one day before receiving radiation treatment); other reasons for reviewing the need for ART included significant loss of neck tissue (6/39, among which 4/6 subsequently underwent ART); significant shrinkage of lymph nodes (8/39, among which 4/8 subsequently underwent ART), noted change of body contour or neck contour (5/39, all of which subsequently underwent ART), significant change in neck position/MVCT scan showed twisting of neck (3/39, among which 1/3 subsequently underwent ART); cord displacement larger than 3mm found on 2D X-ray film when Linear Accelerator was used for treatment (1/39, among which no ART was performed subsequently); teeth removal (1/39, among which no ART was performed subsequently). Some patients exhibited more than one of the above reasons for reviewing the need for ART.

Among the 16 patients who subsequently underwent ART after being review, 4 (out of 16) patients received ART due to unfit of their thermoplastic casts, probably due to significant BW loss and shrinkage of neck lymph nodes; 5 (out of 16) were found to have insufficient dose coverage of their neck node regions, probably due to significant change in neck contour and/or shrinkage of neck lymph node and/or significant loss of neck tissue and/or change of body contour; 4 (out of 16) were found to have significant increase in high dose area over neck skin, probably due to significant decrease in neck lymph node volume/ neck tissue volume; 2 (out of 16) were found to be risky in the dose tolerance of spinal cord (a portion of spinal cord was going to receive >45Gy, the 45Gy isodose line was in close proximity to the spinal cord), probably due to significant change in neck contour; 1 (out of 16) was found to have slightly overdosed optical chiasm, possibly due to change of body contour caused by significant BW loss; 1 (out of 16) received ART because a part of the contoured target

volume was found to be outside the body contour. Some patients exhibited more than one of the above reasons for receiving ART.

MRI scanning protocols

The MR images acquisition parameters were as follows: axial T2-w using short-tau-inversion-recovery (STIR) MR sequence (repetition time [TR]/ echo time [TE]: 7640/97 ms, field-of-view [FOV] = 24 x 24 cm, number of acquisition = 1, slice thickness = 4 mm x 25 slices, spacing: 0.75mm x 0.75mm x 4.4mm, matrix: 320) and axial CET1-weighted spin-echo MR sequence (repetition time [TR]/ echo time [TE]: 739/17 ms, field-of-view [FOV] = 24 x 24 cm, number of acquisition = 1, slice thickness = 3 mm x 48 slices, spacing: 0.938mm x 0.938mm x 3.3mm, matrix: 256).

Classes of extracted features

The distribution and names of extracted features, including gray level co-occurrence matrices (GLCM), gray level dependence matrices (GLDM), gray level run-length matrices (GLRLM), gray level size zone matrix (GLSZM), and neighboring gray tone difference matrices (NGTDM) categories, can be found in Supplementary Table 1 and Figure 2.

Supplementary Table S1. Distribution of extracted features in current study.

	**Distribution of Extracted Features (n = 479)**				
	**Original Features**	**LoG-derived Features (Kernel size:2mm)**	**LoG-derived Features (Kernel size:3mm)**	**LoG-derived Features (Kernel size:4mm)**	**LoG-derived Features (Kernel size:5mm)**
Shape	14	0	0	0	0
First-order	18	18	18	18	18
GLCM	24	24	24	24	24
GLDM	14	14	14	14	14
GLRLM	16	16	16	16	16
GLSZM	16	16	16	16	16
NGTDH	5	5	5	5	5
**Sub-Total**	**107**	**93**	**93**	**93**	**93**

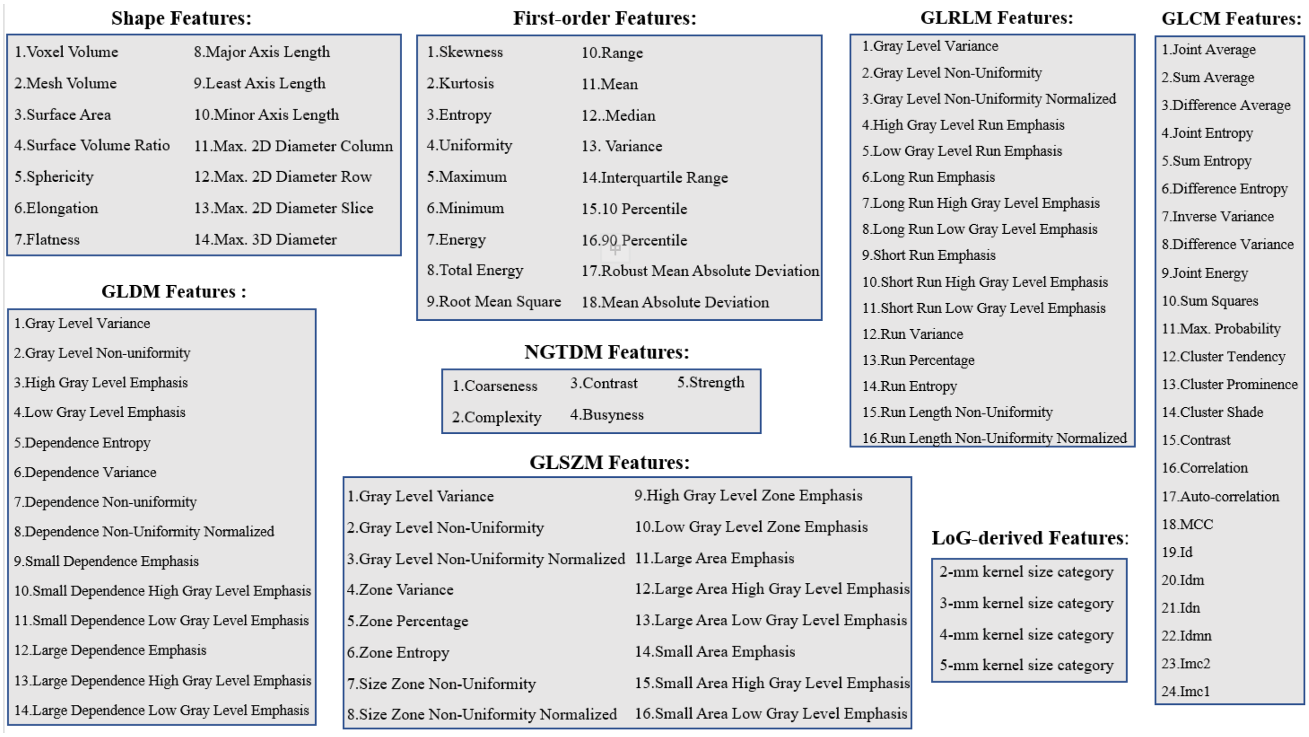

Supplementary Figure S2. Names of radiomic features extracted in this study.

Feature screening methodology

Part I: The goal of part I was solely to eliminate the radiomic features (out of the 53 features) that were less, if not least, robust. In part I (a), we put all the re-planned patients (n=13) plus other 17 non-re-planned patients into training cohort (n=30) with a three-fold cross-validation, and performed multiple rounds of training to generate a total of 400 sub-models. Then, we ranked the 53 features in a descending order according to their frequency of occurrence (from a maximum of 400 to a minimum of zero) to obtain the first set of features. In part I (b), we put all 70 patients into the training cohort with a ten-fold cross-validation, and generated 400 sub-models for the same goal. Then, we ranked the 53 features as described to obtain the second set of features. Following this, we then eliminated the features that did not appear in any of the 400 sub-models, as they were considered as the least robust to our outcome prediction. After all these, we compared both sets of features and reduced to 28 features according to their frequency of occurrence in all the models.

Part II: The goal was to further eliminate the less predictive features by considering five different possible distributions of re-planned patients (n=13) in training and validation cohorts. We put 51 patients (including 8 re-planned patients) into training cohort, and remaining 19 patients (including 5 re-planned patients) into validation cohort. We employed three-fold cross-validation to generate 200 sub-models, we then ranked the 28 features in a descending order according to their frequency of occurrence (from a maximum of 200 to a minimum of zero). We repeated the above procedures with other 4 ratios of number of re-planned patients in training cohort to that in validation cohort (i.e. 9:4, 10:3, 11:2, and 12:1). Subsequently, by comparing these 5 sets of features, we further reduced the features number to 16.

Part III: The goal was to create an optimized radiomic model with reduced number of remaining features. We put 51 patients (including 8 re-planned patients) into training cohort, and remaining 19 patients (including 5 re-planned patients) into validation cohort with three-fold cross-validation. Two thousand sub-models were generated, each of them might contain different amounts of features. We then categorized the 2,000 sub-models according to the numbers of remaining features in these models and evaluated these categories one-by-one. By assessing the consistency and stability of area under the receiver operator characteristic (ROC) curve (AUC) in training cohort among different sub-models in specific category, we further removed features that appeared to be less influential to the AUC values and hence less predictive to the outcome.
